# Cancer diagnosis in areas of conflict

**DOI:** 10.3389/fonc.2022.1087476

**Published:** 2022-12-22

**Authors:** Akram Al-Ibraheem, Ahmed Saad Abdlkadir, Ali Mohamedkhair, Miriam Mikhail-Lette, Mohammad Al-Qudah, Diana Paez, Asem H. Mansour

**Affiliations:** ^1^ Department of Nuclear Medicine, King Hussein Cancer Center, Amman, Jordan; ^2^ School of Medicine, The University of Jordan, Amman, Jordan; ^3^ Nuclear Medicine and Diagnostic Imaging Section, Division of Human Health, Department of Nuclear Sciences and Applications, International Atomic Energy Agency, Vienna, Austria; ^4^ Department of Microbiology and Pathology - Faculty of Medicine - The Hashemite University, Zarqa, Jordan; ^5^ Department of Diagnostic Radiology, King Hussein Cancer Center, Amman, Jordan

**Keywords:** cancer diagnosis, imaging, war, conflict, refugees, Middle East, resources

## Abstract

To date, many Arab countries within the Middle East are facing political, financial, and social instability from war and conflicts. These conflicts have led to severe resources shortages and sometimes complete breakdowns in cancer care and diagnosis. Cancer diagnosis at early stages is the most vital step in achieving optimal cancer care and outcomes. Shortages in cancer diagnostic services have meant that many people within areas of conflict are ultimately deprived of these services in their own countries. Therefore, many of these cancer sufferers must bear travel expenses to neighboring countries in order to seek these services. A lack of prevention, screening, and diagnostic services for this population is known to deepen the cancer care deficit within these areas. Additionally, the financial burden of traveling abroad alongside the need to secure childcare and time off work can be overwhelming. As a result, patients within areas of conflict are frequently diagnosed at later stages and are less likely to receive optimal management plans. Though conflict-affected regions encounter many similar challenges in delivering quality cancer care, pronounced region-specific differences do exist. Therefore, it is important to build a roadmap that can provide tailored solutions to deficits in instruments, manpower, and facilities for each and every region involved. Keeping in mind the importance of collaboration and coordination on national and international levels to address the ground disparity in cancer diagnostic services, the main objective of this review article is to examine the significant problems, shortages, and difficulties in providing cancer diagnosis with a focus on imaging to conflict-affected populations in the Middle East (mainly Iraq, Syria, Yemen, and Sudan). Finally, we discuss how access to cancer diagnostic imaging services has been impacted by these conflicts.

## 1 Introduction

Critical cancer diagnostic care, including the synergistic benefit of combined diagnostics and appropriate treatments, has been adversely impacted by conflicts within many Arab countries. At the time of conflicts, many hospitals, centers, or private sector providers were destroyed, rendered non-functional, or variably compromised (1). Concurrently these sites experienced a shift of focus toward military services, with less stable financial support for health services. These and other factors have contributed to a regression in achieving developmental milestones for cancer diagnostic services.

The burden of cancer diagnosis extends beyond the affected nations to many of their bordering neighbors, compelled to rally additional efforts to provide healthcare services insofar as possible. Many cancer patients, including refugees, cannot afford the costs of cancer diagnosis and are not covered by insurance companies. This can lead to significant, often catastrophic health expenditures for these patients and their families. Common to this patient population are cancer diagnoses at advanced stages, too late to hope for cures. Life-prolonging and/or palliative management can be considered. Meanwhile many others are forced to endure their cancers without any interventions offered or received.

In parallel fashion, cancer care has been significantly impacted by conflicts and wars (2). This was reflected in the lack of access to optimal cancer surgery, radiation therapy, and chemotherapy services, as well as cancer diagnosis (2). Long-term conflicts are known to paralyze many aspects of cancer care, including demands, supplies, equipment, infrastructure, and personnel (1). Many cancer care seekers are forced to travel abroad for better cancer care delivery (3). The majority of cross-border cancer care seekers will end up experiencing financial and economic hardship in addition to the psychological burden of the disease itself (3).

The outbreak of war led to the termination of training programs in many countries, largely due to a lack of experienced personnel. Iraq is one of the countries where nuclear medicine training has ended due to the conflict in the region. In addition, many advanced diagnostic services are still not available in Arab countries that have been affected by war. The departure of many scientists, physicians, and physicists in search of a safer way of life has constituted a major barrier. Staff members who have remained currently deal with a wide range of issues, including a lack of modern services, a lack of local support networks, and a lack of steady financial support. Revision plans must be developed to improve diagnostic services in conflict zones. The goal of the international oncologic community should be to optimize cancer diagnostic and therapeutic services. Potential advantages of this optimization strategy include early cancer detection and establishing cultural and economic solutions to enhance the overall plan (4). This would require cooperation between societies, associations, and private cancer institutions.

The primary goal of this review article is to discuss difficulties experienced during conflicts in Arab countries (mainly Iraq, Syria, Yemen and Sudan). Addressing the known barriers and concerns in cancer diagnosis, with a particular focus on cancer imaging capability. Considering elaborating on comparable and nation-specific issues. Finally, develop a revival strategy for every country in order to offer the best diagnostic care possible.

## 2 Cancer diagnosis in Iraq

Iraq has experienced significant hardship due to various wars, sanctions, and embargoes. The significant impact of multiple wars, the 2003 invasion, and subsequent conflicts have severely affected the delivery of cancer diagnostic services. Since 1980, overall local healthcare delivery has declined remarkably. A possible link has been drawn between the use of depleted uranium-tipped ammunition and white phosphorus munitions in warfare and the development of cancer in Iraqi cancer patients (5). Over the last four decades, the government has been more interested in the security issues by nourishing its military service than in caring for medical demands. Simultaneously, cancer incidence increased by 50% during the last decade (6). The high incidence of cancer, coupled with inadequate healthcare infrastructure, is definitively impeding cancer diagnosis and treatment services (7). In order to revive cancer diagnostic services, a plan must be put in place to address and circumvent all known problems. It’s also necessary to establish a lasting infrastructure in order to control cancer on both local and global scales (8).

### 2.1 The current status of manpower in Iraq

There are relatively few qualified doctors, technicians and scientists in Iraq for cancer diagnosis. The lack of manpower is known to have a severe impact on diagnostic service capacity. The number of experts able to provide accurate pathologic diagnoses for childhood cancers is very limited, and they are often very busy (9). There is an insufficient number of female radiologists specialized in breast imaging. Similarly, there is a severe shortage of male radiologists in the field of breast imaging. This is further complicated by patient conservatism and religious fervor that are known to paralyze the delivery of this essential service. The number of such highly specialized specialists, such as radiation oncologists, interventional radiologists, and pediatric onco-radiologists, is also inadequate (**Table 1**). Other specialties, such as nuclear medicine (NM) physicians and technicians, as well as endoscopists, are primarily found in tertiary hospitals and large cities (**Table 1**). One of the valid solutions to this problem is to build a local network of specialists with similar scientific interests. This would help physicians to work together more effectively and efficiently and make it easier for new training programs to be adopted. The result, a new generation of specialists would emerge and hopefully fill the relevant gaps.

**Table 1:** Manpower facts and problems in Iraq.

Specialty	Amount of manpower	Known problems
Paediatric oncology	10	- Limited numbers - Busy schedules
Radiologists	570	- Defect in female radiology service - Limited interventional radiologists - Inadequate numbers of paediatric onco-radiologists
Nuclear medicine	21	- Limited numbers of board-certified nuclear physicians
Nuclear technologists	6	- Inadequate numbers of nuclear technologists
Endoscopists	35	- Sub-optimal numbers

### 2.2 Infrastructures, supplies, demands, and systematization

The use of diagnostic imaging services is essential for correctly diagnosing, staging, and monitoring the treatment and progression of cancers. As well, imaging is used to guide procedures such as the placement of ports (central venous catheters) for systemic administration of medicines per evidence-based protocols. Imaging also diagnoses complications or co-morbidities, such as pneumonia. In Iraq, cancer diagnostic services would benefit from systematic reviews, especially in the public sector. After the Gulf War, material shortages became more evident (10). The imposed sanctions have banned a range of cancer services, including linear accelerators, PET machines, some chemotherapy drugs, and radioisotopes (11). In order to restrict Iraq’s capability to manufacture weapons of mass destruction, the country experienced a 13-year deficit in cancer diagnosis. During that timeframe, many Iraqi cancer patients were forced to travel abroad to access advanced diagnostic services. One of the main challenges is the lack of availability of equipment for diagnostic imaging such as mammography, endoscopy, and pediatric imaging. The lack of equipment was evident for some time and was accompanied by a 50% cancer incidence surge in the last decade (6), which made the problem worse (**Table 2**). During wars, the contamination resulted from depleted uranium and other toxic wastes are among justified observations for the resultant cancer diagnosis to incidence discordance (12). For example, there are a maximum of 180 CT units and 125 MR units in the country. These values translate to only 4.5 and 2.3 unites per million people (13). These values are still insubstantial when plotted against the capacity of services achieved in neighboring countries like Saudi Arabia which has achieved values of about 25.1 and 15.8 respectively (14). A recent microsimulation model by Ward et al. suggested that countries with few imaging units could benefit significantly from optimizing and expanding their resources (15). It is important to note that the biggest improvement in cancer diagnostic care would come from improving MRI and CT equipment (15).

**Table 2:** Current status of diagnostic services among Iraqi Provinces.

KurdistanIncluding (Erbil, Sulaymaniyah and Duhok)		
X-ray units	234	
Ultrasound units	92	
CT units	15	
MR units	8	
Gamma Camera units	3	
PET/CT units	3	
Rest of IraqIncluding (Baghdad, Mosul, Basra, Dhi Qar, Babil, Anbar, Diyala, Kirkuk, Salah Al-Din, Najaf, Wasit, Qadisiya, Karbala, Maysan, and Muthanna)		
X-ray units	1585	
Ultrasound units	1356	
CT units	158	
MR units	98	
Gamma Camera units	11	
PET/CT units	9	
Total number of resources		Units per million
X-ray units	1819	45.5
Ultrasound units	1448	36.2
CT units	173	4.3
MR units	106	2.6
Gamma Camera units	14	0.35
PET/CT units	12	0.3
Current Observation		
Severe limitations are currently observed in nuclear medicine field followed by conventional imaging (CT and MR units). Future vision should provide effective plan to invest in these two major services to fortify cancer diagnostic service delivery in the country.		

To date, many public sector hospitals have relied on physical archiving for patients’ data. However, this method is becoming increasingly outdated and inefficient. Physical archiving is considered to be the least secure and most primitive method of archiving. A large number of data sets previously stored physically are either missing or difficult to recover, and many more were destroyed during previous conflicts. Electronic information systems, such as Picture Archiving and Communication Systems (PACS), Radiology Information Systems (RIS), or Health Information Systems (HIS), are needed to store and access patient data easily.

### 2.3 IRAQI training program: Achievements and drawbacks

During the time of conflict in 1985, the Iraqi training program was established (9). The goal of this program is to meet the increased demand for expert professionals in various fields. The program has been successful in providing specialists for many medical and diagnostic branches for several years. There are several advanced diagnostic fields that are, however, not addressed in the current plan, including molecular cytogenetic immunopathology, next generation sequencing (NGS) training, and nuclear medicine (NM) board program. These fields lack senior specialists able to adopt the new training programs. Noteworthy and promising, the Iraqi Ministry of Health has implemented a program to address the identified problems in coordination with the Iraqi Board for Medical Specialties. This resulted in the establishment of a board-certified radiotherapy training program to address the shortage in this field (16).

### 2.4 The current status of the nuclear medicine service in Iraq

The NM field had provided the most advanced diagnostic services among all neighboring countries up until 1980. Multiple NM governmental centers in large provinces provided diagnostic and therapeutic services (17). Before 1990, Iraq relied on local sources of radioiodine to meet the needs of each nuclear medicine center. After the establishment of sanctions, the country was no longer able to produce radioiodine and became reliant on international suppliers (**Table 3**). The war also had a profound impact on the Iraqi training program for NM. The training program for NM specialists was stopped in 1996, resulting in a limited number of specialists in the following years. To date, many of the available specialists in NM have been forced to complete their residency and training programs in Jordan to gain the experience required to serve their country. There is a high demand for NM technicians, but there is a shortage of qualified professionals. A concurrent lack of radio-pharmacists and radiochemists exists, likely due to a lack of certified education and training programs in these disciplines. There is not yet an established local society for NM in Iraq. NM practice in 2003 was limited by a lack of equipment, with only 5 gamma cameras and no SPECT/CT or PET/CT available (**Table 3**). This translates into a ratio of 0.2 units per million people (13). Despite increasing investment in infrastructure, Iraq is still ranked among the least-resourced Arab countries in terms of the number of equipment units per million people. The current number of SPECT/CT devices, gamma cameras, and PET/CT machines is still not adequate, at only 0.4 units per million (13). Regions that are free from conflict usually have much better infrastructure delivery. For example, Kuwait and Jordan have a total of 10.3 and 2.0 per million, respectively (18).

**Table 3:** Table demonstrating the effects of UN Sanctions on Nuclear Medicine services and program in Iraq.

Items	Before Sanction	During Sanction	After Sanction
**No. of Gamma camera units**	5	0	+7
**No. of PET/CT units**	0	0	+14
**No. of Private sectors providing nuclear medicine services**	0	0	+11
**RAI^131^ Supply Source**	Local	Prohibited	Imported
**Nuclear Medicine training Program**	Established	Stooped	Not Adopted

### 2.5 Overcoming the triple: Non-homogeneity, bungling, and workforce shortages

**Figure 1:**
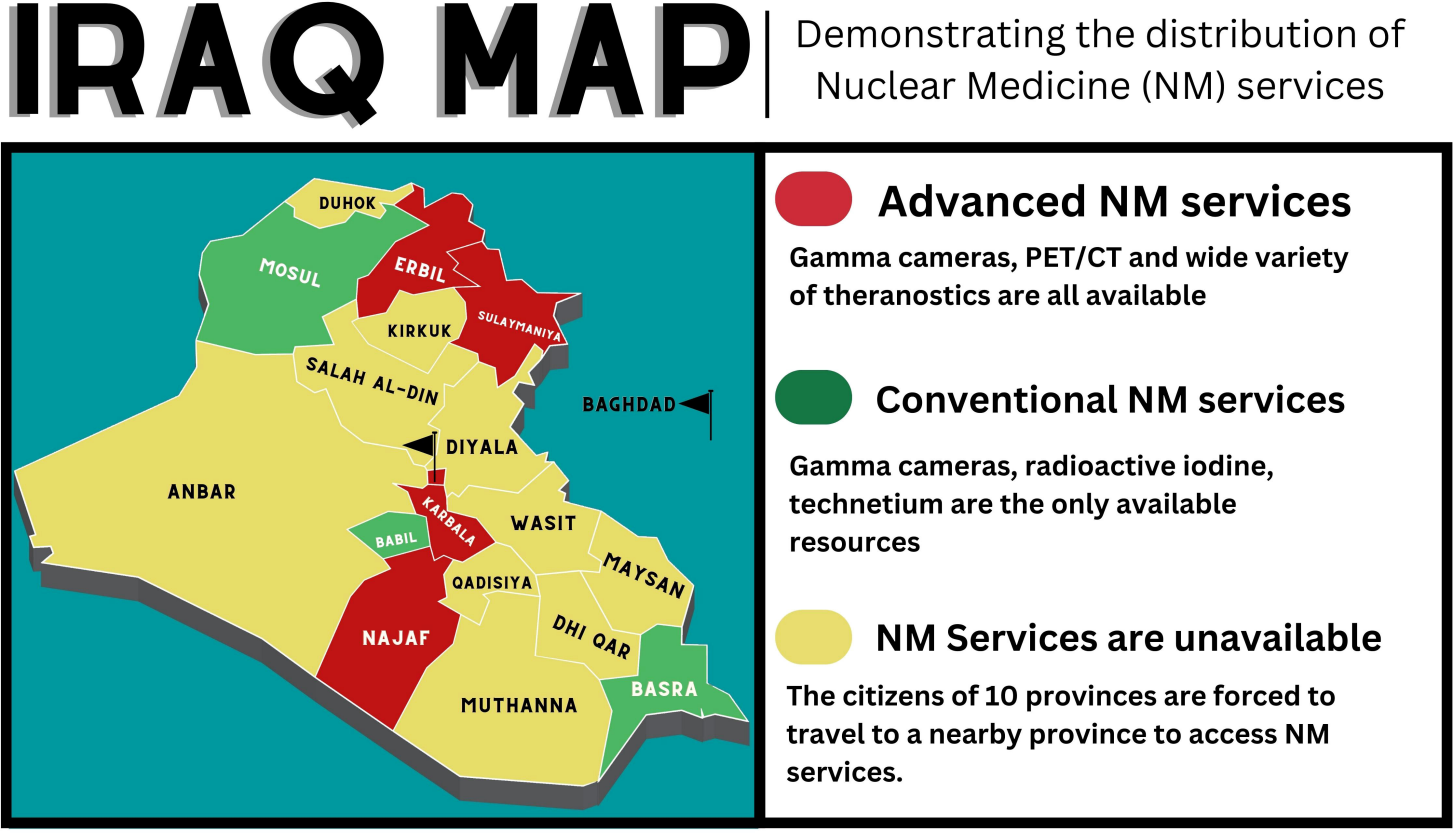
The distribution of NM services among Iraqi provinces.

The war against Islamic State in Iraq and Sham (ISIS) has left some of Iraq’s provinces (i.e., Mosul) with destroyed health care centers. Many of Mosul’s hospitals were severely damaged during the conflict and are currently not operational. At the same time, many of the southern provinces were involved in mismanagement of resources, which deprived locals of access to cancer diagnostic services. In addition, the lack of certain medical specialties and subspecialties is evident in many rural areas and major provinces. The current map of cancer diagnostic services in Iraq shows a non-uniform distribution (**Figure 1**). The current distribution of cancer care services in Iraq implies that residents of the majority of northern provinces, along with a few southern provinces as well as western provinces, have to travel to Baghdad, Erbil, Karbala, or Najaf to access the latest services in cancer diagnosis. Additionally, most of the advanced centers and hospitals are located within the private sector (7). These hospitals are increasingly offering their services without any insurance program to cover the expenses (7). This trend is likely to continue as the costs of health care continue to rise. Many patients will experience financial hardship in order to access current medical services. To ensure cancer diagnostic services are effective, it is necessary to overcome the challenges mentioned above. Assessing and strategically prioritizing service needs, based on population size, infrastructure, and manpower deficits, are vital to achieving the best possible patient outcomes.

### 2.6 The current impact of addressed problems on cancer diagnosis

There is a substantial disparity in the quality of cancer diagnostic services available in Iraq, when compared to its more stable neighboring countries. It has been observed that Iraq is one of the Middle Eastern nations wherein cancer incidence has increased significantly despite inadequate cancer diagnosis (6). During times of war and turmoil, Iraq is unable to maximize its potential in the development of its diagnostic and therapeutic sectors. This problem is further complicated by the lack of security and financial instability that are known to hugely impact cancer diagnosis.

## 3 Cancer diagnosis in Syria

In Syria, prior rapid progress in cancer diagnostic capacity was halted and has regressed considerably since the outset of civil war. As a result of the conflict, several previously active diagnostic fields have suffered shortages of personnel, destruction of infrastructure, or neglect. The economic constraints and shift in focus to military and weaponry fields have left many medical and oncologic services underdeveloped. The departure of many well-known scientific figures, specialists, and physicists from the country was driven by them seeking a more stable environment. A comprehensive and long-term solution is necessary to effectively address cancer diagnosis in Syria.

### 3.1 The current status of manpower in Syria

The findings of a recent study indicate that many diagnostic cancer departments are facing significant shortages, neglect, and workload issues (19). The problem of an imbalanced workload/workforce has become increasingly prevalent in recent years, particularly in the aftermath of the war. The unavailability of many specialized medical services has had a detrimental effect on besieged areas. According to recent surveys given to cancer specialists, only Damascus among the seven regions offers preventive measures such as mammograms, colonoscopies, and pap smears (**Table 4**) (20). In East Aleppo, there are no oncologists or pathologists to serve the population of 500,000 people (20). There is a lack of trained technicians in the field of nuclear medicine, which includes medical physicists, specialists in hybrid imaging, and radiopharmacists/radiochemists (**Table 5**). This lack of trained personnel limits the advancement of nuclear medicine technology. This is especially the case for PET/CT, where there are only a few specialists and technologists.

**Table 4:** Summary of available infrastructures for cancer diagnosis in Syria. Modified from the source “Cancer Care in Times of Crisis and War: The Syrian Example, Table 2”.

Resources	Major Provinces	Besieged Provinces
Pathology	Available	Limited*
Conventional Imaging	Available	Limited
Mammography	Limited	Extremely limited*
Nuclear Medicine Services	Limited	Not available
Screening	Not available	Not available
Genetic testing	Not available	Not available
Clinical Trials	Not available	Not available
Teleradiology	Not available	Not available

*Limited: Services that are clustered in city capital of all besieged provinces but lacking in peripheral areas. Resources capabilities, infrastructure and manpower are suboptimal in these areas.

*Extremely limited: Services that are confined to city capital of only few provinces but lacking in many other provinces. Resource capabilities, infrastructure and manpower are suboptimal in these areas.

**Table 5:** Past and current status of manpower and infrastructure in Syria demonstrating pattern of slow growth, no growth and degradation in certain resources and personnel during war.

Resource	Total Number of Diagnostic units			Observation
	Before War	Current Status	Current units per million	
X-ray	992	1001	58.8	Slow Growth
CT	297	300	17.6	Slow Growth
MRI	152	152	8.9	No growth
SPECT	4	7	0.4	Slow Growth
PET/CT	2	2	0.12	No growth
Mammography	200	207	12.1	Slow Growth
Cyclotron	1	1	0.06	No growth
Personnel	Total Number of available Staff in charge			Observation
	Before War	Current Status		
NM Physicians	11	5		Degradation
NM Technologists	12	11		Degradation
Medical Physicists	24	25		Slow Growth
Radiologists	98	73		Degradation
Future Recommendation				
The current observation necessitate investment in nuclear medicine field to ensure even and adequate delivery of this vital service. Additionally, revival plan must be created to account for current problems in each and every department.				

### 3.2 Infrastructure, supplies, demands, and systematization

Since the conflict in 2011, Syria has been under economic sanctions that have had a negative impact on its cancer diagnostic system (**Table 4**). Sanctions prevented the import of essential supplies, such as non-domestic manufactured radioisotopes and equipment parts, preventing important cancer diagnostic equipment from being repaired. All clinics currently have access to basic diagnostic tools, such as laboratory blood tests, biopsies, and basic imaging studies (20). There is a lack of access to conventional imaging modalities in besieged areas as compared to major cities (**Table 4**). Positron emission tomography (PET) scanners are only available in Damascus, and patients have to pay for them (12). To date, advanced cancer diagnostic services, such as genetic testing and interventional radiology, are not available in many areas (20). These vital services are currently not adopted and remained unexplored in both pre-conflict and post-conflict times. Patients who can afford to pay for advanced services are often referred to neighboring countries for care. But many cancer patients cannot afford to travel or pay for expensive treatment, so they do not receive the care they need. The lack of funding creates difficulties in managing equipment, including maintaining it properly and ensuring its quality. There is a lack of PACS, RIS, or HIS. Currently, teleradiology is unavailable due to insufficient IT infrastructure (PACS, RIS, HIS).

### 3.3 Syrian refugees: A new problem since the war

**Figure 2:**
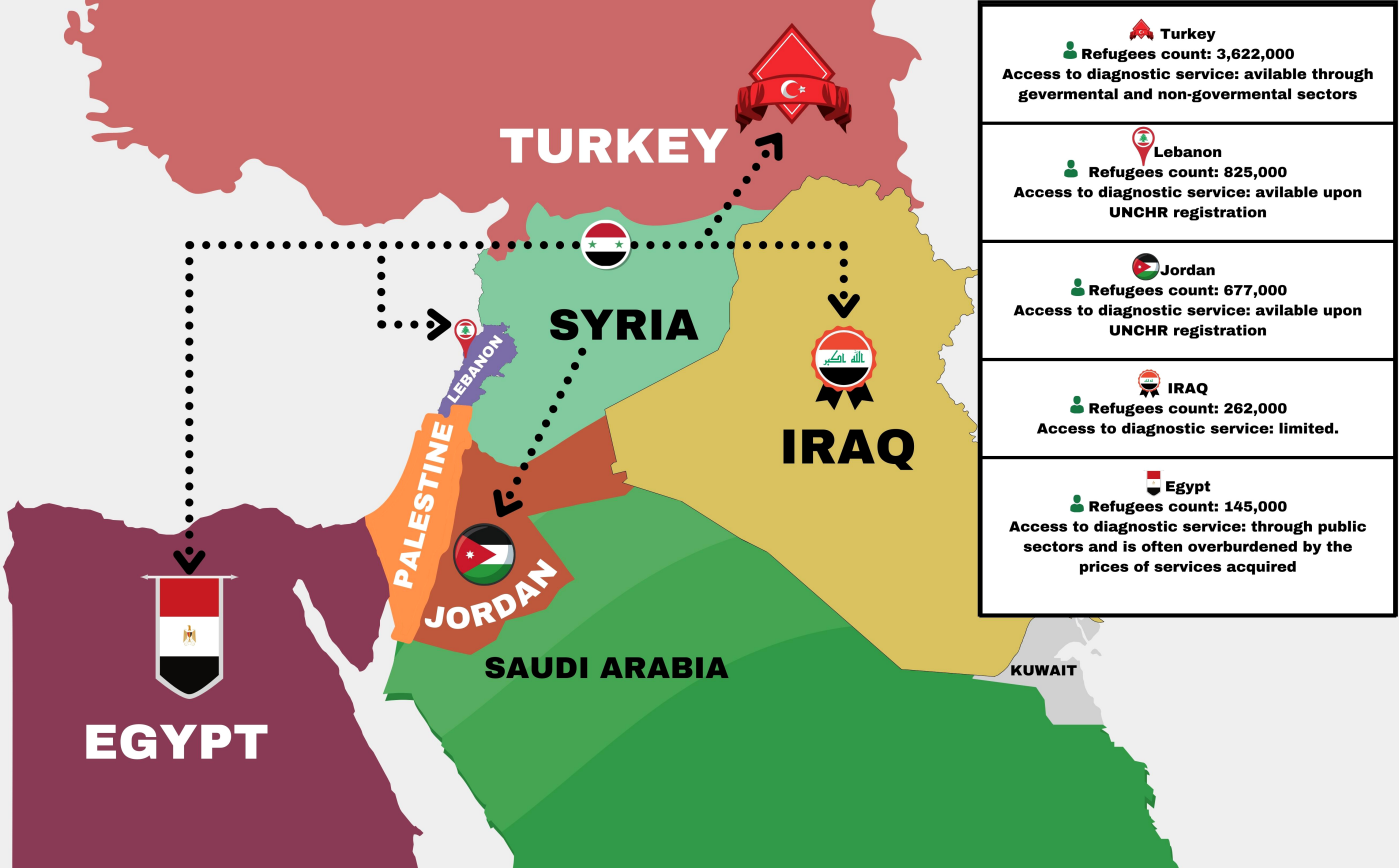
The current situation of Syrian refugees in surrounding nations in terms of numbers and access to services for cancer diagnosis. Modified from the Source “Syria Regional Refugee Response 2022”.

The Syrian conflict has resulted in the displacement of millions of Syrians, who have fled to neighboring countries like Turkey, Iraq, Jordan, Egypt, and Lebanon (**Figure 2**) (21–26). The Syrian refugee crisis has also led to an influx of refugees into Europe, with many making the dangerous journey across the Mediterranean Sea to countries like Greece, Germany, and Sweden in search of safety and refuge. Many host countries have experienced financial difficulties as a result of providing cancer diagnostic care for many cancer sufferers among refugees. The King Hussein Cancer Center (KHCC) in Jordan has reported that a total of 356 Syrian refugees have received full cancer care (27). The King Hussein Cancer Foundation Goodwill Fund was responsible for paying all patients’ costs, which amounted to around USD $11 million (27). Many Syrian refugees and cancer patients are currently accessing cancer diagnostic services at KHCC (27). The Nuclear Medicine Department at KHCC has been providing advanced cancer diagnostic and therapeutic services since 2013 (28). Additionally, Abdul-khalek et al. carried out a broad-range population study on the total cost of cancer diagnostic and therapeutic services among Syrian refugees in neighboring countries (29). This study found that the total cost of cancer diagnosis and treatment services for refugees was approximately 140.23 million euros (29). There are many Syrian refugees who have not had access to cancer diagnostic services. It is important for countries that host cancer patients to cooperate with charities and other organizations that can provide financial assistance. Making this change will ease the financial burden for both cancer patients and the countries hosting them.

### 3.4 The current impact of addressed problems on cancer diagnosis

When assessing the current status of cancer diagnosis in Syria, one can discern a division of the country into two major subgroups. The country is currently divided into besieged areas and governmental areas. The major cities and provinces are currently under the control of the government, while other cities are not (**Table 4**). The disparities in the provision of cancer diagnosis between the two groups have been attributed to the consequences of these conflicts (20) (**Table 4**). At present, regions under regulation are finding it challenging to meet requirements, though they may have attained triumph in particular disciplines or areas. Conversely, areas under siege are deprived of major diagnostic services, either unavailable or severely restricted. In response, effort must be duplicated to account for this non-uniformity that’s known to affect cancer diagnosis in this country.

## 4 Cancer diagnosis in Yemen

Yemen is facing significant challenges in terms of political unrest and economic underdevelopment. According to a recent study, nearly two-thirds of the population are living in poverty, and over half are food-insecure (30). Collectively, the population is struggling to advance itself in an increasingly globalized world. This has had a significant impact on patients’ ability to pay for cancer diagnostic services, making cancer care and overall healthcare facilities less affordable (31). According to the latest estimates, Yemen has the lowest health spending in the world, as well as in the Middle East (32). This is a major problem for the country, as it strives and toils to provide adequate healthcare for its citizens. Cancer diagnostic deficits are caused by a variety of problems that persist today.

### 4.1 The current status of infrastructure in Yemen

The seven-year conflict in Yemen has had a significant adverse effect on the nation’s health sector, especially in terms of cancer treatment. Cancer patients experience difficulties in accessing basic cancer diagnostic services as well as comprehensive cancer care. Cancer patients in Yemen face two main challenges: their personal battle against cancer and the fight to get access to cancer diagnostic and therapeutic services. The cancer diagnosis rate in Yemen is significantly lower than average due to the extremely low number of cancer imaging equipment, especially evident in NM and MR imaging (33). As a result, cancer incidence is greatly underestimated and does not reflect the true picture of cancer trends on a national level. According to data, there are currently fewer than 100 CT scanners, 30 MRI units, and 50 mammography devices in use (33). NM is least of all diagnostic services in terms of existing equipment numbers with only 5 gamma cameras available locally (0.2 units per million people) but unfortunately, they are predominantly nonfunctional due to lack of manpower and radiopharmaceuticals. It is noteworthy that Yemen is the only Arab country that does not provide advanced nuclear diagnostic services such as SPECT, SPECT/CT or PET/CT, which are not available in the country (18).

### 4.2 The burden of financial instability

Currently, the only local cancer centers that provide most of the diagnostic services required for many Yemeni cancer patients are located in Hadramout and Sana’a. Established in 2013, the National Oncology Center in Hadramawt is a non-profit cancer center known for providing cancer diagnostic and treatment services (30). Even though the center plays a vital role, the salaries of the working team are not always covered by the government. This is a problem that needs to be addressed in order to ensure that the center can continue to function properly (30). The organization’s working staff are financially dependent on donations from charitable foundations and small-town organizations in order to maintain the operating budget and offer nonprofit services (30). This dependence means that the center is limited in its ability to provide services and meet its operating budget (30). The center has recently faced significant challenges in continuing to provide free services due to the political unrest in Yemen, the shifting economic climate, and the lack of external funding (30). This refers to clinics, chemotherapies, medications, lab work, and mammograms. Because it doesn’t have enough money, the center will have to shut down and stop giving its services (30). The lack of health insurance reflects lower access to care, and the financial burden is known to limit the patient’s adherence to cancer diagnosis and care (34). This is why cancer sufferers are often diagnosed at advanced stages.

### 4.3 The effects of war on nuclear medicine centers

Prior to the outbreak of war in Yemen, nuclear medicine services were already inadequate. Through that time, only primitive nuclear medicine tests were carried out through the use of gamma cameras, which were the sole operational units in the nation. Yemen’s conflict has had a significant impact on NM services and centers. Despite the fact that the country has a sufficient number of nuclear medicine physicists, the country’s lack of NM physicians has resulted in the discontinuation of limited and primitive NM diagnostic services. According to a recent report, the import of radioisotopes has been discontinued at Al-Thawra Hospital in Sana’a, rendering the gamma camera useless (33). The other nuclear medicine department in Aden has been unable to provide any services since they failed to install a gamma camera. Yemen doesn’t have enough high-tech medical imaging services, so many people, who can afford it, have to leave the country to get care.

### 4.4 Seeking treatment abroad

The lack of financial stability and qualified medical staff, as well as the absence of necessary diagnostic and therapeutic options, have forced many cancer sufferers to travel abroad (33). Cancer patients often receive care at a later stage when the disease is more advanced. Patients who cannot afford to travel for medical care are not getting optimal care in their home country. Providing financial stability, and progressively establishing new basic and advanced healthcare facilities of high standards, are lofty goals; but these may be the best initial infrastructural steps toward sustainably improving oncologic healthcare for the Yemeni people.

### 4.5 The current impact of addressed problems on cancer diagnosis

Cancer diagnosis, incidence, and care in Yemen have been undervalued since the outbreak of conflict. Financial instability, personnel departure and insecurity have complicated the access to cancer diagnosis. Many patients are seeking treatment and care outside the country in hopes of obtaining a more effective treatment plan. The provision of cancer services is often suboptimal, resulting in an impediment to effective cancer diagnosis and treatment.

## 5 Cancer diagnosis in Sudan

Sudan, officially the Republic of Sudan, is a country in Northeast Africa. It shares borders with the Central African Republic to the southwest, Chad to the west, Egypt to the north, Eritrea to the northeast, Ethiopia to the southeast, Libya to the northwest, South Sudan to the south, and the Red Sea to the east. It has a population of about 45 million people as of 2022 and occupies 1,8 million square kilometers, making it Africa’s third-largest country by area, and the third-largest by area among Arab countries (35). It was the largest country by area in Africa and the Arab countries until the secession of South Sudan in 2011, since which both titles have been held by Algeria (36). Its capital is Khartoum, and its most populous city is Omdurman (part of the metropolitan area of Khartoum).

### 5.1 The past and current status of overall diagnostic services in Sudan

Sudan ranked among the first African countries to forge ahead in the areas of diagnosis and treatment of cancer. The first comprehensive cancer center opened there in the early 1960s, the Radiation and Isotope Centre in Khartoum (RICK) (37). Thereafter Sudan initiated establishment of their first National Cancer Registry (NCR) in 1967 (36). Unfortunately, currently there is no active national cancer registry in the country. However, Sudan, like the rest of the world, has seen an increase in the incidence of cancer over the last few decades (38). In March 2021, the Global Cancer Observatory (GLOBOCAN of the WHO/International Agency for Research on Cancer (IARC) estimated 27,382 new cancer cases among both sexes in the year 2020 in the capital of Sudan (Khartoum) compared to 6,771 new cases in 2010 (39, 40).

### 5.2 Current status of manpower and infrastructure

**Figure 3:**
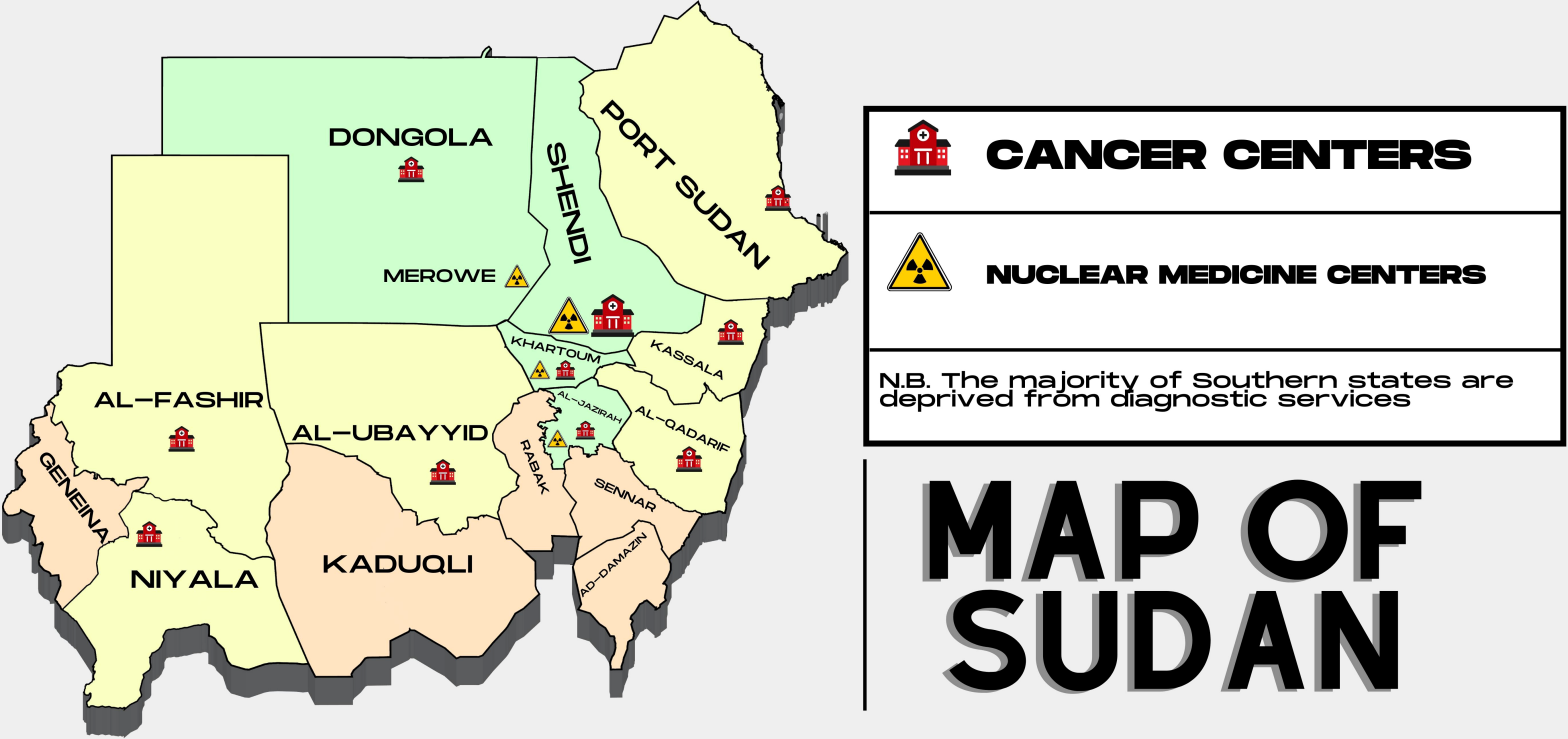
The availably of cancer diagnostic service providers in Sudan.

To date, there are fourteen (governmental and private) health facilities that provide cancer care in Sudan (**Table 6**). Of these, only three governmental facilities provide comprehensive cancer care in Sudan, and they are located in the center of the country. Others either lack at least one major diagnostic/treatment discipline (pathology, radiology, NM, radiotherapy) or provide only chemotherapy-based anticancer treatment. Furthermore, although there are national training programs in some of the aforementioned cancer-related specialties, there is still a tangible lack of professionals and personnel dealing with the significant rise in cancer incidence. This is superimposed upon the fact that well-qualified and experienced staff leave the country for better prospects abroad. As well, because of the political and economic instability, there is a lack of a comprehensive national vision to address such shortcomings in this health sector. A compounding factor is the impact of sanctions imposed on Sudan following the takeover of a prior regime in power, which has affected the reach of state-of-the-art equipment and software in the field. Currently, there are a total of seven (active) NM centers in the country (governmental and private) (**Table 7**). Interestingly, only two centers established outside the capital city provide conventional nuclear diagnostic services through available SPECT devices (**Figure 3**). On the other hand, the remaining five NM centers are all situated in the capital city and offer radioactive nuclides like technetium and fluorine. Since there are no cyclotrons, reactors, or generators in the country, all of the radioactive materials are brought in from other countries. To put this shortage of diagnostic services in context, and when comparing NM services in neighboring countries, Egypt for example, there are 88 PET/CT centers in the country, 55 in Cairo alone, 11 in Alexandria, and 22 centers in the rest of the country. Not to mention the presence of 3 Cyclotrons, 1 PET/MR and 3 PEM units in Cairo, in addition to a radiopharmaceutical factory that covers 60% of the country needs from radiopharmaceuticals.

**Table 6:** Treatment facilities in Sudan in the governmental and private sectors. Adapted from the Source “Cancer Care in Arab World, Chapter 16, General Oncology Care in Sudan, Table 16.3”.

No.	Name of the facility	Established	Location	Sector	Services
1	Khartoum Oncology Hospital (RICK)	1962	Khartoum state Capital of Sudan	Governmental	- Radiation - Nuclear medicine - medical oncology services
2	National Cancer Institute	1997	Wad Madani city Second city of Sudan Middle Sudan	Governmental	- Radiation - Nuclear medicine medical oncology services
3	Tumor Therapy and Cancer Research—Shendi	2008	Nile Valley state North Sudan	Governmental	- Radiation - Nuclear medicine medical oncology services
4	Khartoum Oncology Specialized Center	2010	Khartoum state Capital of Sudan	Private	Medical oncology services
5	Khartoum Breast Care Center	2010	Khartoum state Capital of Sudan	Private	Medical oncology services
6	Dongola Cancer Center	2012	North state North Sudan	Governmental	Medical oncology services
7	Taiba Cancer Center	2013	Khartoum state Capital of Sudan	Private	Medical oncology services
8	Shafi Specialized Oncology Center	2015	Khartoum state Capital of Sudan	Private	Medical oncology services
9	East Oncology Center	2015	Qadarif state East Sudan	Governmental	Medical oncology services
10	Port Sudan Oncology Center	2015	Red Sea state East Sudan	Governmental	Medical oncology services
11	Kordofan Cancer Center	2015	North Kordofan state West Sudan	Governmental	Medical oncology services
12	Merowe Oncology Center	2017	North state North Sudan	Governmental and Private	Radiation Nuclear medicine medical oncology services
13	Nyala Cancer Center	2019	South Darfur state West Sudan	Governmental	Medical oncology services
14	Al-Fashir Cancer Center	2020	North Darfur state West Sudan	Governmental	Medical oncology services
15	Universal Hospital Sudan	2021	Khartoum state Capital of Sudan	Private	Radiation Nuclear Medicine Medical oncology Services

**Table 7:** Nuclear medicine centers in Sudan in the governmental and private sectors.

No.	Name of the facility	Location	Sector	Equipment	Workforce
1	Khartoum Oncology Hospital (RICK)	Khartoum state Capital of Sudan	Governmental	SPECT SPECT/CT	18
2	National Cancer Institute	Jazirah state Second city of Sudan Middle Sudan	Governmental	2 SPECT	8
3	Tumor Therapy and Cancer Research—Shendi	Nile Valley state North Sudan	Governmental	SPECT	5
4	Alneelain Center	Khartoum state Capital of Sudan	Private	SPECT	10
5	Ahmed Gasem center	Khartoum state Capital of Sudan	Private	SPECT	3
6	Royal Care Hospital	Khartoum state Capital of Sudan	Private	SPECT	4
7	Universal Hospital Sudan	Khartoum state Capital of Sudan	Private	PET/CT SPECT/CT	11
8	Merowe Oncology Center	North state North Sudan	Governmental and Private	SPECT	0

Since its independence, Sudan has endured a series of violent conflicts and political instability in terms of being the country with the most coup attempts on the continent, with a tally of 17 attempts (6 of which led to a successful takeover of power). Conflicts started with the civil war in the southern part of the country (ended by the secession of South Sudan) at the east-central borders with Ethiopia and Eritrea, and finally the war in Darfur, west of the country. As a result, the country went through decades of decline in infrastructure and a lack of basic supplies of water and sanitation, power, and transportation in areas of conflict. This situation has produced a humanitarian crisis that burdens not only the areas of conflict but the whole country. For example, the internally displaced people (IDPs) from the region of Darfur are estimated to be around 3 million as of January 2022, according to the United Nations High Commissioner for Refugees (UNHCR). For these reasons, the health services, including cancer diagnosis, in the regions of conflict as well as in the relatively peaceful neighboring regions have been significantly affected as an inevitable sequela. For example, there is only one area in the west of the country with radiology services, one pathology lab, and no NM services, even though about 9 million people reside there.

### 5.3 The lack of cancer diagnostic services

Prior to 2003, when the Darfur conflict began, there was no cancer care in the region at all. However, as **Table 6** shows, the region has recently seen the opening of multiple centers that provide at least one chemotherapy clinic with an on-site oncologist. Nevertheless, the other services related to diagnosis and treatment are still missing in an area yet to cope with the aftermath of the conflict and the lack of basic services as explained above. The case is not quite different in other areas of the country where multiple states still lack well-established pathology labs and NM services, and all patients are sent to the capital insofar as possible.

When we look at the radiology services, we find that there are only 6 mammography units in the whole country in just two regions (Khartoum “5 machines” and Wad Madani “1 machine” cities). 17 CT machines in Khartoum the capital, 6 in Wad Madani city and 1 device in all of Senar, Kasala, Gadarif, Merowe, Kosti, Halfa, Shendi, Alobied and Tangesi cities. 10 MRI machines in the capital, 3 in Wad Medani and only one machine in rest of the country found in Merowe.

Currently all radiopharmaceuticals are imported from outside the country with no domestic production. Furthermore, theranostic applications are limited to I^131^ therapeutic clinics, provided in only two centers in the country. These clinics are not open on a regular basis, with interrupted services due to logistic issues, including that all radioactive iodine supplies are also brought from outside the country.

### 5.4 The current impact of addressed problems on cancer diagnosis

Regarding cancer care services, all services in governmental centers are provided either free of charge or with only minimal fees through a national insurance company. However, not all services are available; some services need to be done in the private sector or outside the country. For example, immunohistochemistry services are exclusively offered in private sector while PET/CT imaging is currently unavailable locally. All of these factors and when compared to neighboring countries depict how of an impact war can produce on the health care system and to the economy in general.

## 6 The vital role of international oncologic community in prosperity of cancer diagnosis and care delivery

Local, Arabic and international communities is one of the established programs to provide solution to many addressed problems in regards to cancer diagnosis. Establishment of local societies for each and every field in cancer diagnosis is vital to achieve optimal harmonization and outcome. Arabic-wise, the amalgamation of many cancer care providers under the umbrella of the pediatric oncology east and Mediterranean group (POEM) have been witnessed in the last decade. This group is currently interested in providing solution and coordination between Arab countries. Internationally, collaboration between the union for international cancer control (UICC) and the World Health organization (WHO) are already underway to address all the barriers encountered in conflict affected regions and beyond (41).

## 7 Conclusion

The damage to the infrastructure and loss of many cancer diagnostic field workers will continue to impede the ability to rebuild a comprehensive cancer diagnostic system after the conflicts have resolved in the aforementioned featured countries. The ability to understand the long-term effects of war is complicated by a lack of communication, the departure of scientific figures, and the discontinuation of training programs and research. There are local and global efforts to mitigate the harm and destruction associated with impaired cancer diagnostic and treatment capacities. In order to create an effective revival program, a clear plan must be developed that identifies obstacles and establishes a roadmap for addressing each issue, one at a time and in a sustainable, phased approach.

## Author contributions

AA-I and ASA conceived the concept, collected data and wrote the manuscript, AM contributed to data collection and along with the other authors listed made substantial, direct intellectual contributions to the work, and approved it for publication.
